# The impact of protein source and grain inclusion on digestibility, fecal metabolites, and fecal microbiome in adult canines

**DOI:** 10.1093/jas/skad268

**Published:** 2023-08-09

**Authors:** Stephanie D Clark, Clare Hsu, Sydney R McCauley, Maria R C de Godoy, Fei He, Renee M Streeter, Emily G Taylor, Bradley W Quest

**Affiliations:** BSM Partners, Bentonville, AR 72712, USA; Department of Animal Sciences, The University of Illinois at Urbana-Champaign, Urbana, IL 61801, USA; BSM Partners, Bentonville, AR 72712, USA; Department of Animal Sciences, The University of Illinois at Urbana-Champaign, Urbana, IL 61801, USA; Department of Animal Sciences, The University of Illinois at Urbana-Champaign, Urbana, IL 61801, USA; BSM Partners, Bentonville, AR 72712, USA; BSM Partners, Bentonville, AR 72712, USA; BSM Partners, Bentonville, AR 72712, USA

**Keywords:** animal-protein, digestibility, fecal microbiome, grain-free, pet food, pulses

## Abstract

This study was conducted to determine the effect of animal protein inclusion rate and grain-free or grain-inclusive diets on macronutrient digestibility, fecal characteristics, metabolites, and microbiota in mixed-breed hounds and Beagles. Four experimental extruded kibble diets were made with varying amounts of animal protein and carbohydrates: 1) high animal protein, grain-inclusive (**HA-GI**), 2) low animal protein, grain-free (**LA-GF**), 3) low animal protein, grain-inclusive (**LA-GI**), and 4) high animal protein, grain-free (**HA-GF**). Thirty-two Beagles and 33 mixed-breed hounds were assigned to 1 of the 4 treatment groups in a completely randomized design that lasted 180 d. All diets were similar in chemical composition and well-digested by the animals. In general, for fecal metabolites, mixed-breed hounds had a greater concentration of total short-chain fatty acid (**SCFA**) and ammonia and lower indole concentration than Beagles (*P* < 0.05). In mixed-breed hounds, LA-GF had a greater (*P* < 0.05) total SCFA concentration than HA-GI and LA-GI; however, this was not observed in Beagles. There were greater concentrations of ammonia, phenol, and indole in HA-GI than in LA-GF (*P* < 0.05). Breed-affected fecal primary bile acid (**BA**) concentration, as mixed-breed hounds had a greater concentration of cholic acid (**CA**) than Beagles (*P* < 0.05). Mixed-breed hounds fed LA-GF resulted in greater CA concentrations than HA-GI and LA-GI (*P* < 0.05). Dogs who consumed LA-GF had lower fecal secondary BA content than the other groups (*P* < 0.05). The distribution of the fecal microbiota community differed in LA-GF compared with the other groups, with lower α-diversity. However, dogs fed LA-GF had the largest difference in composition with greater Selenomonadaceae, Veillonellaceae, Lactobacillaceae, *Streptococcus*, *Ligilactobacillus*, *Megamonas*, *Collinsella aerofaciens*, and *Bifidobacterium* sp. than the other groups. A significant breed effect was noted on nutrient digestibility, fecal metabolites, and microbiota. A treatment effect was observed in LA-GF as it resulted in greater fecal SCFA, lower protein fermentative end products, greater fecal primary BAs, lower fecal secondary BA concentrations, and shifts in fecal microbiota.

## Teaser

A grain-free diet with low animal protein can cause changes in fecal characteristics, metabolites, and microbiota in dogs compared with traditional grain-inclusive and/or high animal protein diets.

## Introduction

Traditionally, animal protein and grains have been the main ingredients used in formulations of commercial pet foods. However, with the diversification of the industry and modern trends, plant-based protein and grain-free diets have become prevalent options in the pet food market (Laflamme et al., 2014; Quest et al., 2022). The protein quality of plant-based ingredients can vary due to source, formulation, and ­processing conditions and methods (Carciofi et al., 2009). Indeed, several studies have noted good protein quality in plant-based diets with high nutrient digestibility (Bednar et al., 2000; Brown et al., 2009; Carciofi et al., 2009). Plant ingredients can also serve as a fiber source and contribute to hindgut fermentation. With pulses, specifically, studies have shown that grain-free diets resulted in greater proportions of fecal primary bile acids (**BAs**) compared with grain-inclusive diets or animal protein-based diets (Pezzali et al., 2020; Reilly et al., 2021a). Research has also supported the theory that a diet’s macronutrient content can shift the gut microbiota (Pilla and Suchodolski, 2021). As gut microbiota have been related to the health status in dogs (Wernimont et al., 2020; Félix et al., 2022), microbiota and the related metabolites have become important parameters for evaluating the effects of nutrition as well.

Fecal metabolites, such as ammonia, indole, and phenol, may have adverse metabolic effects (Oliphant and Allen-Vercoe, 2019). The primary source of ammonia in the intestinal tract is due to the bacterial degradation of the protein (Weber and Veach, 1979). Moreover, diets high in indigestible proteins are fermented by the microbiota in the colon, resulting in other compounds like indoles and phenols (Birkett et al., 1996). The concentration of ammonia and these by-products are highly indicative of the animal’s diet and unique gastrointestinal microbiota. For instance, greater concentrations of ammonia, indoles, and phenols have been found in the fecal matter when ingesting high levels of animal protein (Cummings and Macfarlane, 1991; Birkett et al., 1996). Furthermore, an overproduction of indole may lead to chronic kidney disease (Meijers and Evenepoel, 2011; Oliphant and Allen-Vercoe, 2019). However, dietary fibers and other non-digestible carbohydrates have been shown to have a positive influence on the microbiota. Short-chain fatty acid (**SCFA**) production can increase when diets are high in fiber, increasing microbial metabolic activity and contributing to normal bowel function. Conversely, ammonia can inhibit the production of SCFA, leading to the assumption that excess ammonia in the diet could be detrimental to animal health (Oliphant and Allen-Vercoe, 2019). In addition, branched-chain fatty acids (**BCFA**) are produced by the microbiome, in the hindgut, and are commonly saturated fatty acids with a methyl branch on one or more carbon chains. Past studies have observed a change in BCFA that are produced by the microbiome, pending the fiber source in the diet (Donadelli et al., 2019). Moreover, a previous study observed an increase in protein fermentation increased the production of BCFA (Xu et al., 2017).

The amount of fat in the diet can directly affect BAs. Diets high in fat induce a discharge of primary BA from the gall bladder. Around 90% to 95% of the primary BAs are absorbed in the distal ileum for further processing in the liver; however, the remaining primary BAs serve as substrates for microbial metabolism. They can then undergo biotransformation to secondary BAs, which can cause colon inflammation (Zeng et al., 2019).

Plant ingredients have also been found to impact the canine fecal microbiota (Sandri et al., 2019; Ciaravolo et al., 2021; Reilly et al., 2021b). Furthermore, in humans, research has been conducted on the correlation between the gut microbiome and the cardiac function (Tang et al., 2019; Chaikijurajai and Tang, 2021). In dogs, only a few studies have investigated the relationship between microbiota and heart diseases and the results have been inconclusive (Yu et al., 2018; Seo et al., 2020; Li et al., 2021; Araki et al., 2022).

Based on published literature, there has been no definitive scientific evidence, thus far, to support causation or a possible link between grain-free diets, animal protein inclusions, and dilated cardiomyopathy (**DCM**) (Mansilla et al., 2019; Freid et al., 2021). However, there have been different hypotheses about how grain-free diets could be linked to DCM, such as dietary fiber causing taurine loss or poor absorption of precursors and lower carnitine and sulfur-containing amino acid content in plant-based diets (Story and Kritchevsky, 1978; Johnson et al., 1998; Mansilla et al., 2019). Given these concerns about poor digestibility between pulse-rich diets in comparison to low pulse inclusion diets, the objective of the current study was to evaluate the effect of animal protein inclusion rate and grain-free diets on macronutrient digestibility, fecal characteristics, metabolites, and microbiota in both mixed-breed hounds and Beagles. It was hypothesized that all diets would be well tolerated and digested, and dogs fed low animal protein diets (i.e., plant-protein rich diets) would have a greater concentration of metabolites from saccharolytic fermentation and shifts in gut microbiota that would support gut fermentation and metabolic activity in comparison to high-animal protein source diets.

## Materials and Methods

### Animals

Pet Food Solution Inc. (Auxvasse, MO) Animal Care and Use Committee approved all animal care procedures before animal experimentation (Study Number: 020098.001). All methods were performed per the United States Public Health Service Policy on Humane Care and Use of Laboratory Animals. This study was part of a larger project evaluating multiple biometric parameters including complete blood count, serum chemistry, plasma, whole blood, skeletal and endomyocardial tissue amino acids, echocardiographs, thyroid and cardiac biomarkers, and physical examinations. Sixty-five intact adult dogs were included in the study: 32 Beagles (16 males and 16 females, mean age 24.2 ± 7.1 mo, mean body weight [**BW**] 9.8 ± 1.5 kg, mean body condition score [**BCS**] of 5.2 ± 1.3) and 33 mixed-breed hounds (16 males and 17 females, mean age 13.3 ± 0.8 mo, mean BW 27.6 ± 3.3 kg, mean BCS 5.3 ± 1.4). With a completely randomized design, dogs were stratified into treatment groups based on breed, sex, and BCS. All dogs were housed in individual cages and randomly assigned to 1 of the 4 housing rooms. Cages were stainless steel with self-spanned polyvinyl chloride-coated expanded metal flooring. All dogs had a photoperiod of 12:12 (L:D) h. Extruded diets were weighed and recorded for each feeding. All dogs were fed once daily (8:00 a.m.) to maintain ideal BCS (5 out of a 9-point scale), and clean water was provided ad libitum.

### Diets

Before the start of formulation, the raw ingredients were tested for complete nutrient profiles before formulation and finished product testing was conducted on all four diets. All diets were extruded in the same manufacturing facility using an extruder screw speed of 350 to 480 rpm, four cooking temperatures through the extruder zones (92.9, 93, 95.8, and 100.5 °C, respectively), and a dryer temp of 107.2 °F. Four experimental diets were made with different inclusion levels of animal-based protein and carbohydrates. High animal protein diets had 70% of their protein content coming from animal sources while low animal protein diets had 45% of the protein in the diet coming from animal protein sources. The diets were 1) high animal protein grain-inclusive (**HA-GI**), 2) low animal protein grain-free (**LA-GF**), 3) low animal protein grain-inclusive (**LA-GI**), and 4) high animal protein grain-free (**HA-GF**). The diets were formulated to meet the Association of American Feed Control Officials (**AAFCO**) nutrient profile for adult canine maintenance. The diets were also formulated to be consistent in macronutrient composition with common ingredients used in pet foods (Tables 1 and 2).

**Table 1.** Ingredient inclusion rates of treatment diets containing different sources of dietary proteins and carbohydrates

Item, % as-is	Dietary treatments^1^			
	HA-GI	LA-GF	LA-GI	HA-GF
Whole yellow corn	22.8	—	30.0	—
Red winter wheat	22.8	—	13.8	—
Corn gluten meal	2.7	—	13.8	—
Sorghum	9.6	—	6.6	—
Beet pulp	3.0	—	3.5	—
Cellulose	1.0	—	1.0	—
Whole green lentil	—	20.8	—	1.0
Yellow pea flour	—	20.8	—	2.0
Pea starch	—	19.5	—	26.9
Dehydrated potato flake	—	1.5	—	26.8
Pea protein	—	3.3	—	—
Pea fiber	—	2.5	—	4.6
Chicken fat	8.2	10.1	9.0	9.1
Chicken meal (low ash)	13.7	2.1	—	16.2
Chicken meal (regular ash)	11.5	7.5	16.5	5.5
Dried chicken	—	6.4	—	3.7
Natural flavor	2.5	2.5	2.5	2.5
Iodine	0.7	0.9	0.8	0.8
Tricalcium phosphate	—	0.8	—	—
Dicalcium phosphate	—	—	0.6	—
Potassium chloride	0.5	0.6	0.6	—
Salmon oil	0.3	0.5	0.3	0.3
Vitamin and mineral premix^2^	0.4	0.4	0.4	0.4
Mixed tocopherols	0.2	0.1	0.2	0.2
Choline chloride	0.1	0.1	0.1	0.1

^1^HA-GI, high animal protein grain-inclusive; LA-GF, low animal protein grain-free; LA-GI, low animal protein grain-inclusive; HA-GF, high animal protein grain-free.

^2^Vitamin E supplement, vitamin A supplement, niacin supplement, d-calcium pantothenate, thiamine mononitrate, riboflavin, pyridoxine hydrochloride, folic acid, vitamin B12 supplement, vitamin D3 supplement, copper proteinate, iron proteinate, manganese proteinate, selenium yeast, zinc proteinate, sodium chloride, magnesium proteinate.

**Table 2.** Chemical composition of treatment diets containing different sources of dietary proteins and carbohydrates

Item	Dietary treatments^1^			
	HA-GI	LA-GF	LA-GI	HA-GF
Dry matter, %	90.7	89.0	89.9	89.6
	% DM basis			
Organic matter	93.6	92.1	92.6	93.9
Ash	6.4	7.9	7.4	6.1
Acid-hydrolyzed fat	16.6	15.5	14.0	17.2
Crude protein	29.5	28.3	30.4	29.7
Total dietary fiber	15.6	15.0	15.1	16.5
Soluble dietary fiber	5.3	5.0	5.0	5.7
Insoluble dietary fiber	10.3	10.0	10.1	10.8
Gross energy, kcal/g	5.3	5.2	5.2	5.3

^1^HA-GI, high animal protein grain-inclusive; LA-GF, low animal protein grain-free; LA-GI, low animal protein grain-inclusive; HA-GF, high animal protein grain-free.

### Chemical analyses and apparent total tract digestibility

Total feces were collected during a 4-d period, on days 176 to 180. Weight and fecal scores were recorded for each fecal sample according to Nestle Purina’s Fecal Scoring Chart on a 7-point scale, then stored at −20 °C until analyzed to determine macronutrient apparent total tract digestibility (**ATTD)**. Additionally, a composite fecal sample from the 4 d for each dog was dried in a 57 °C forced-air oven. Once dried, fecal samples were ground through a 2-mm screen using a Wiley mill (model 4, Thomas Scientific, Swedesboro, NJ) and ­analyzed in duplicate according to methods described below under “Chemical Analyses”.

Experimental diets and dried fecal samples were ground through a 2-mm screen using a Wiley mill (model 4, Thomas Scientific). Dry matter (**DM**), ash, organic matter (**OM**), acid-hydrolyzed fat (**AHF**), crude protein (**CP**), total dietary fiber (**TDF**), and gross energy (**GE**) of the diet and fecal samples were determined. DM and ash were determined according to AOAC (2006; methods 934.01 and 942.05). Total nitrogen values were determined using 6.25 a conversion value, according to AOAC (2006; method 992.15) with CP calculated from Leco (TruMac N, Leco Corporation, St. Joseph, MI). AHF was analyzed according to AACC (1983) and Budde, (1952). GE was determined through bomb calorimetry (Model 6200, Parr Instruments Co., Moline, IL). TDF was analyzed according to Prosky et al. (1992).

Total fecal samples were collected during the last 4 d of the experimental period (Meineri et al., 2021). All fecal samples were weighed, scored, and stored at −20 °C until analyzed to determine macronutrient **ATTD** using the ­formula:


$$\begin{array}{l} ATTD=\frac{\begin{array}{lll} & (food\;intake\;in\;grams\;on\;DM\;basis\;*\;\;\%\;nutrient\;in\;the\;diet)-\; & (fecal\;output\;in\;grams\;on\;DM\;basis\;*\;\;\%\;nutrient\;in\;feces)\; \end{array}}{\left(food\;intake\;in\;grams\;on\;DM\;basis\;*\;\;\%\;nutrient\;in\;the\;diet\right)} \end{array}$$

The same calculation was used to determine digestible energy based on analyzed GE of the diet and fecal samples.

### Fecal collection, preparation, and fermentative metabolite and BA analyses

A fresh fecal sample was collected from each dog within 15 min of defecation on days 0, 30, 90, and 180. Aliquots of the fresh fecal sample were collected for analyzing BCFA, SCFA, ammonia, phenols and indoles, BAs, and microbiota. Fecal samples allocated for fermentative end-product analysis were stored at −20 °C. Fecal samples allocated for BA and microbiota were stored at −80 °C until analyses.

Three grams of fresh fecal samples were collected into bottles (Nalgene, Rochester, NY) containing 3 mL of 2 N hydrochloric acid for BCFA, SCFA, and ammonia analyses. Concentrations of SCFA and BCFA were measured using gas chromatography according to (Sunvold et al., 1995). Fecal ammonia concentrations were determined according to Chaney and Marbach, (1962). An additional 2 g of feces was collected and placed into tubes for phenol and indole analyses. Fecal phenol and indole concentrations were analyzed through gas chromatography according to Flickinger et al., (2003). BA analysis was performed according to Batta et al. (2002) to measure unconjugated cholic acid (**CA**), chenodeoxycholic acid (**CDCA**), lithocholic acid (**LCA**), deoxycholic acid (**DCA**), and ursodeoxycholic acid (**UDCA**). Fecal samples were lyophilized for 1 wk, pulverized using a spatula (Smart Spatula, USA Scientific, Ocala, FL), and stored at −20 °C until analysis. Aliquots of 10 to 15 mg of lyophilized feces were weighed into disposable glass centrifuge tubes (5 mL, Kimble-Chase, Rockwood, TN). A total volume of 200 μL of butanol containing 0.25 mg/mL of internal standards, undecanoic acid, was added to each fecal sample. Forty μL of HCl (37% American Chemical Society reagent) were added and vortexed for 30 s. Samples were then incubated at 65 °C for 4 h. After incubation, samples were dried under nitrogen gas at 65 °C. Two hundred microliters of TMS-derivatization agent (Supelco’s Sylon HTP HMDS + TCMS + Pyridine, 3:1:9 Kit) were added to the sample and incubated for an additional 30 min at 65 °C. The sample was again dried under nitrogen gas at 65 °C for 25 min. Samples then were resuspended in 300 μL of hexane, vortexed briefly, and centrifuged for 10 min at 3,200 × *g*. The supernatant was then transferred to a GC/MS vial insert (300 μL glass with polymer feet, Agilent, Santa Clara, CA) and capped. A Thermo TRACE 1310 Gas Chromatography coupled with ISQ LT single quadrupole Mass Spectrometer was used for all separations. The GC/MS used a capillary column (DB-1ms Ultra Inert, Agilent, Santa Clara, CA) using the following dimensions: 30 m, diameter: 0.250 mm, film thickness: 0.25 μm. A 20:1 split was used after a 1-μL sample was injected with an inlet temperature of 280 °C. After injection, the oven temperature was held at 50 °C for 1 min, then ramped at 20 °C per minute to a final temperature of 300 °C and held for 12 min. Helium was used as the carrier gas at a nominal flow rate of 1.5 mL/min.

### DNA extraction, amplification, sequencing, and bioinformatics

Total DNA was extracted from fresh fecal samples using a Mo-Bio PowerSoil kit (MO BIO Laboratories, Inc., Carlsbad, CA). Quantification of DNA concentration was completed using a Qubit 2.0 Fluorometer (Life Technologies, Grand Island, NY). A Fluidigm Access Array (Fluidigm Corporation, South San Francisco, CA), combined with Roche High Fidelity Fast Start Kits (Roche, Indianapolis, IN), were used to amplify the 16S rRNA gene. Full-length 16S PacBio (Pacific Biology, Menlo Park, CA) specific primers, forward (AGRGTTYGATYMTGGCTCAG), and reverse (RGYTACCTTGTTACGACTT) tags, were added per the PacBio protocol. The quality of the amplicons’ regions and sizes was confirmed by Fragment Analyzer (Advanced Analytics, Ames, IA). A DNA pool was generated by combining equimolar amounts of the amplicons from each sample. The pooled samples were selected by size on a 2% agarose E-gel (Life Technologies) and extracted using a Qiagen gel purification kit (Qiagen, Valencia, CA). The pooled, size-selected, and cleaned products were then run on an Agilent Bioanalyzer to confirm the appropriate profile and mean size. The Roy J. Carver Biotechnology Center at the University of Illinois performed PacBio sequencing. The 16S amplicons were generated with the barcoded full-length 16S primers from PacBio and the 2x Roche KAPA HiFi Hot Start Ready Mix (Roche, Willmington, MA). The amplicons were pooled and converted to a library with the SMRTBell Express Template Prep kit 2.0 (Pacific Biology). The library was sequenced on 1 SMRTcell 8M in the Sequel II using the CCS sequencing mode and a 10hs movie time.

Sequences were analyzed using DADA2 (version 1.14; Callahan et al., 2016). This analysis imported amplicon sequence variants (**ASV**) into the phyloseq R package (McMurdie and Holmes, 2013). Sequences were agglomerated based on the distribution of inter-taxa phylogenetic distances independent of a reference database, using a threshold of 0.175. A total of 216 taxa were determined. Prevalence filtering was performed after agglomeration; ASV was only retained for further analysis if they were observed in 4 or more samples. After singleton filtering, the number of assigned taxa was reduced to 157.

Bray–Curtis dissimilarity (Bray and Curtis, 1957), UniFrac distance (Hamady et al., 2010), and weighted UniFrac distance (Hamady et al., 2010) were calculated between samples after converting ASV abundances to proportions. Nonmetric multidimensional scaling (**NMDS**) was then used to visualize the distance matrices in two dimensions. Alpha diversity was assessed by observed taxa, Chao1, and Inverse Simpson indexes. The DESeq2 R package (Love et al., 2014) was used to identify differentially abundant taxa between treatments; a false discovery rate (**FDR**; Benjamini and Hochberg, 1995) lower than 0.05 were used to declare statistical significance.

### Statistical analyses

Data were analyzed by the PROC MIXED procedure in SAS (SAS Institute, Inc., version 9.4, Cary, NC). The model was run with a fixed effect of diet and a random effect of sex. Effects over time were investigated using repeated-measures analyses. Based on the Bayesian Information Criterion, compound symmetry covariance structure gave the best fit and was used for all repeated measures analyses. The interaction of treatment, breed, and day and their main effects were reported using a Fisher-protected least significant difference test with a Tukey–Kramer multiple comparison test. When interactions were significant, the slice statement was used to evaluate differences within each simple main effect. Data are represented as the least squares mean ± SEM and considered significant at *P* < 0.05.

## Results

### Diet composition, food intake, fecal output, and ATTD

The macronutrient composition of the dietary treatments is reported on a DM basis (**Table 2**). DM content ranged from 89.0% for LA-GF to 90.7% for HA-GI. Organic matter concentrations among experimental diets were also similar, varying from 92.1% for LA-GF to 93.9% for HA-GF. AHF content was similar among groups, ranging from 14.0% for LA-GI to 17.2% for HA-GF. CP content was comparable among treatment diets, ranging from 28.3% to 30.4%. TDF concentration ranged from 15.0% to 16.5%, with soluble dietary fiber ranging from 4.9% to 5.7% and insoluble dietary fiber from 10.0% to 10.8%. Likewise, GE was similar among diets (5.2 to 5.3 kcal/g).

Data for food intake and fecal output are shown in g/d per kg metabolic BW (**Table 3**), to avoid bias due to significant differences in BW between breeds, which can be the main factor that impacts metabolizable energy requirements and daily food intake. Dogs, from both breeds, among all treatment groups, had similar food intake on an as-is and DM basis. No interaction between treatment and breed was observed for fecal output on an as-is and DM basis, but there was a main effect of treatment. On an as-is basis, fecal output for the LA-GF group was significantly greater than the other treatment groups. Dogs, from both breeds, consuming HA-GF had lower fecal output DM basis than those consuming LA-GF and LA-GI (*P* < 0.05). There was also an effect of breed on fecal output DM basis as Beagles had greater DM output than mixed-breed hounds (*P* < 0.05).

**Table 3:** Mean food intake and fecal output of dogs fed dietary treatments containing different sources of dietary proteins and carbohydrates^1^

	Treatments								SEM^2^	*P*-value		
	Beagles				Mixed-breed hounds							
	HA-GI	LA-GF	LA-GI	HA-GF	HA-GI	LA-GF	LA-GI	HA-GF		Trt	Breed	Trt* Breed
Fecal Score^3^	2.5^c,y^	2.9^ab,y^	2.3^bc,y^	2.8^a,y^	3.1^c,x^	3.8^ab,x^	3.6^bc,x^	4.1^a,x^	0.17	0.0012	<0.0001	0.1558
Item, g/d/kg BW^0.75^												
Food intake as-is	41.4	43.8	38.2	38.4	40.9	46.0	49.7	40.0	3.78	0.4446	0.1821	0.3730
Food intake DM basis	37.5	39.0	34.3	34.4	37.1	40.9	44.7	35.8	3.39	0.4763	0.1822	0.3703
Fecal output as-is	14.7^b^	21.1^a^	17.1^b^	13.7^b^	11.8^b^	21.5^a^	14.0^b^	12.6^b^	1.89	0.0008	0.2282	0.8081
Fecal output DM basis	5.5^ab,x^	5.6^b,x^	6.2^b,x^	3.6^a,x^	3.9^ab,y^	5.4^b,y^	4.6^b,y^	3.4^a,y^	0.51	0.0009	0.0148	0.3535

^1^HA-GI, high animal protein grain-inclusive; LA-GF, low animal protein grain-free; LA-GI, low animal protein grain-inclusive; HA-GF, high animal protein grain-free; Trt, treatment.

^2^Standard error of mean.

^3^Fecal scores: 1, hard, dry pellets; small hard mass; 2, firm, but not hard, pliable, segmented in appearance; 3, moist surface, little or no visible segmentation; 4, very moist, log-shaped, leaves residue on the ground; 5, very moist but has a distinctive shape, leaves reside on the ground; 6, texture but no defined shape, presents as piles or spots; and 7, watery, liquid that can be poured.

^a,b,c^Within a row, treatment means with different superscripts differ *P* < 0.05.

^x,y^Within a row, breed means with different superscripts differ *P* < 0.05.

All diets were well-digested with macronutrient ATTD above 80% (**Table 4**). An interaction among treatment and breed (*P* < 0.05) was observed for DM, OM, AHF, CP, and TDF ATTD. Among all four dietary treatments, mixed-breed hounds fed HA-GF had consistently the greatest (*P* < 0.05) DM, OM, AHF, CP, and TDF ATTD whereas Beagles fed LA-GI had the lowest ATTD. A similar difference (*P* < 0.05) was also observed in digestible energy in which mixed-breed hounds fed HA-GF had the greatest and Beagles fed LA-GI had the lowest. Independent of breed, dogs fed HA-GF had the greatest (*P* < 0.05) digestible energy and ATTD for TDF when compared with all the other groups. Dogs fed HA-GF also showed greater (*P* < 0.05) ATTD for DM and OM than LA-GF and LA-GI. In contrast, lower (*P* < 0.05) ATTD for DM and OM was observed for dogs fed LA-GF and LA-GI in comparison with dogs fed HA-GF. The ATTD of AHF was lowest (*P* < 0.05) in dogs fed LA-GI and greatest in dogs fed LA-GF and HA-GF. The ATTD of CP was lowest (*P* < 0.05) for dogs on LA-GI and greatest for dogs fed HA-GF. TDF ATTD was the greatest (*P* < 0.05) in HA-GF compared to the other three groups.

**Table 4.** Apparent total tract macronutrient digestibility of dogs fed dietary treatments containing different sources of dietary proteins and carbohydrates

Digestibility, %	Treatments^1^								SEM^3^	*P*-value		
	HA-GI		LA-GF		LA-GI		HA-GF					
	B^2^	H^2^	B	H	B	H	B	H		Trt	Breed	Trt × breed
Dry matter	85.3^bc^	89.4^ab^	85.6^abc^	86.7^abc^	81.8^c^	89.1^ab^	89.4^ab^	90.4^a^	1.10	0.0010	0.0001	0.0172
	% DM basis											
Organic matter	88.2^ab^	91.6^ab^	88.0^bc^	89.1^ab^	85.7^c^	91.7^ab^	91.1^ab^	92.0^a^	0.88	0.0043	<0.0001	0.0176
Acid-hydrolyzed fat	93.6^b^	95.5^b^	95.2^b^	95.8^ab^	90.7^c^	94.6^b^	96.1^ab^	96.3^a^	0.57	<0.0001	0.0003	0.0080
Crude protein	86.1^ab^	90.5^a^	86.7^ab^	87.2^ab^	82.5^b^	89.7^a^	88.2^a^	90.3^a^	1.07	0.0218	<0.0001	0.0248
Total dietary fiber	54.9^c^	69.4^ab^	55.4^bc^	61.3^abc^	48.0^c^	70.6^a^	70.0^a^	73.5^a^	3.22	0.0005	<0.0001	0.0175
Digestible energy, kcal/g	4.6^b^	4.8^ab^	4.7^b^	4.7^b^	4.4^c^	4.7^b^	4.9^a^	4.9^a^	0.04	<0.0001	<0.0001	0.0113

^1^HA-GI, high animal protein grain-inclusive; LA-GF, low animal protein grain-free; LA-GI, low animal protein grain-inclusive, HA-GF, high animal protein grain-free.

^2^B, Beagles; H, mixed-breed hounds.

^3^Standard error of mean.

^a,b,c^Superscripts with different letters in a row represent statistical differences (*P* < 0.05).

### Fecal metabolite and BA concentrations

Fecal metabolite concentrations (DM basis) were analyzed and are presented in **Tables 5 and 6** for mixed-breed hounds and Beagles, respectively. A treatment-by-day interaction was observed on days 30, 90, and 180, DM concentration was greater (*P* < 0.05) in HA-GI (30.7%, 30.0%, and 27.4%, respectively) than in LA-GF (25.7%, 24.7%, and 21.8%, respectively) and HA-GF (25.8%, 25.8%, and 21.3%, respectively). However, there was no statistical difference in DM between HA-GI and LA-GI throughout the experimental period (*P* > 0.05). Similarly, an interaction between treatment and day (*P* < 0.05) was also observed for fecal ammonia, phenol, and indole concentrations. Fecal ammonia concentration only differed among treatment groups on day 180 (*P* < 0.05); HA-GI had the greatest concentration (2.53 mg/g) compared with LA-GF (1.83 mg/g), LA-GI (2.10 mg/g), and HA-GF (1.75 mg/g). Fecal phenol concentration only differed among treatment groups on day 90 (*P* < 0.05); HA-GF (1.42 µmol/g) was significantly greater than LA-GF (0.36 µmol/g). Indole concentrations at days 30 and 90 were significantly lower in LA-GF (0.00 µmol/g, 0.15 µmol/g, respectively) in comparison to concentrations at day 30 for HA-GI (0.88 µmol/g), LA-GI (1.15 µmol/g), but not HA-GF (0.34 µmol/g). Diet LA-GF at days 30 and 90 were also lower than HA-GI (1.98 µmol/g), La-GI (1.53 µmol/g), and HA-GF (1.16) on day 90; and HA-GI (1.21 µmol/g) on day 180. (**Tables 5 and 6**).

**Table 5.** Fecal metabolite concentration of mixed-breed hounds fed experimental diets containing different sources of dietary proteins and carbohydrates

Item^1^	Day 0				Day 30				Day 90				Day 180				SEM^2^	*P*-Value					
	HA-GI	LA-GI	HA-GF	LA-GF	HA-GI	LA-GI	HA-GF	LA-GF	HA-GI	LA-GI	HA-GF	LA-GF	HA-GI	LA-GI	HA-GF	LA-GF		Trt	Day	Breed	Trt^*^ Day	Trt^*^ Breed	Day^*^ Breed
Dry matter, %	30.77	32.20	31.93	32.91	30.56^a^	30.29^a^	23.92^b^	23.80^b^	31.53^a^	30.58^a^	24.66^b^	23.62^b^	25.69^a^	26.53^a^	19.54^b^	21.23^b^	1.31	<0.0001	<0.0001	0.022	**<0.0001**	0.572	0.075
Ammonia, mg/g	1.78	1.53	1.69	1.62	0.19	0.14	0.12	0.10	0.21	0.28	0.46	0.39	2.53^a^	2.10^b^	1.75^b^	1.83^b^	0.17	0.048	<0.0001	0.088	**0.010**	0.826	0.546
Phenol and indole, µmol/g																							
Phenol	0.69	0.67	0.82	0.65	0.41	0.43	0.35	0.08	1.23	0.90	1.42^a^	0.36^b^	0.75	0.46	0.53	0.11	0.24	0.103	<0.001	0.371	**0.020**	0.455	0.227
4-Ethylphenol	3.58	2.69	3.18	4.07	1.57	1.67	1.01	0.89	3.54^a^	3.86^a^	2.78^a^	1.18^b^	1.75	2.30	2.09	1.80	0.53	0.029	<0.0001	0.995	**<0.001**	0.700	0.098
Indole	1.51	1.06	1.41	1.15	0.88^a^	1.15^a^	0.34^ab^	0.00^b^	1.98^a^	1.53^a^	1.16^a^	0.15^b^	1.21^a^	0.71^ab^	0.43^bc^	0.12^c^	0.31	<0.0001	<0.0001	0.004	**<0.0001**	0.972	0.992
SCFA^3^, µmol/g																							
Acetate	284.94	246.08^y^	280.96^x^	275.80^x^	361.76	398.02^y^	436.77^x^	467.30^x^	231.68	256.79^y^	306.52^x^	310.52^x^	259.03	262.93^y^	299.28^x^	317.74^x^	24.37	**0.009**	**<0.0001**	**0.023**	0.436	0.122	0.699
Propionate	178.31	162.99	167.78	176.89^†^	191.19^b^	196.71^b^	247.07^b^	338.13^a,†^	140.8^bc^	143.07^c^	194.81^ab^	228.83^a,†^	151.61^b^	143.68^b^	205.72^a^	248.17^a,†^	18.14	<0.0001	<0.0001	<0.0001	**<0.001**	**0.040**	0.657
Butyrate	51.95	41.95	50.78^x^	49.72^y^	53.92	57.07	59.42^x^	45.01^y^	40.66	42.15	56.80^x^	51.49^y^	44.78	51.11	54.23^x^	53.51^y^	6.23	**0.032**	**0.032**	0.757	0.167	0.205	0.873
Total SCFA	515.19^y^	451.01^y^	499.53^x^	502.41^x^	606.86^y^	651.8^y^	743.26^x^	850.44^x^	413.14^y^	442.01^y^	558.14^x^	590.83^x^	455.41^y^	457.72^y^	559.24^x^	619.41^x^	43.89	**<0.001**	**<0.0001**	**0.002**	0.094	0.053	0.833
BCFA^4^, µmol/g																							
Isobutyrate	7.09	8.59	6.15	5.74	7.39^a^	10.62^a^	5.28^ab^	3.75^b^	5.80^a^	5.87^a^	5.65^a^	3.03^b^	8.03	6.56	6.79	6.53	0.99	<0.0001	0.001	0.246	**0.006**	0.051	0.574
Isovalerate	10.12	8.69	9.35	8.51	10.67^a^	11.32^a^	7.50^a^	5.83^b^	8.70^a^	9.14^a^	8.58^a^	5.16^b^	11.05^a^	10.51^ab^	9.19^b^	8.51^b^	1.01	<0.0001	0.017	0.139	**0.015**	0.352	0.318
Valerate	5.56	0.62	1.22	1.84^†^	2.47	2.60	8.04	14.57^†^	4.97^b,*^	7.32^b,*^	9.93^b,*^	28.66^a,*,†^	1.74^b,*^	9.08^b,*^	13.62^b,*^	36.30^a,*,†^	3.29	0.001	<0.0001	0.001	**<0.0001**	**0.007**	**<0.001**
Total BCFA	22.77	17.90	16.71	16.09^†^	20.53	24.54	20.82	24.15^†^	19.47^*^	22.33^*^	24.15^*^	36.84^*,†^	20.81^b,*^	26.14^b,*^	29.59^b,*^	51.35^a,*,†^	4.17	0.486	<0.0001	0.001	**0.002**	**0.024**	**0.010**

^1^Values are on a dry matter basis. Trt * Day * Breed interaction was not significant (*P* > 0.05) for any treatment. HA-GI, high animal protein grain-inclusive; LA-GF, low animal protein grain-free; LA-GI, low animal protein grain-inclusive; HA-GF, high animal protein grain-free.

^2^Standard error of the mean.

^3^Short-chain fatty acids.

^4^Branched-chain fatty acids.

^a,b,c^Within a row, treatment means with different superscripts within each day differ (*P *< 0.05).

^x,y^Within a row, treatment means with different superscripts differ (*P *< 0.05).

^*^Within a row, mixed-breed hounds’ means with different superscripts differ from Beagles within each day (*P *< 0.05).

^†^Within a row, mixed-breed hounds’ means with different superscripts differ from Beagles within each treatment (*P *< 0.05).

**Table 6.** Fecal metabolite concentration of beagles fed experimental diets containing different sources of dietary proteins and carbohydrates

Item^1^	Day 0				Day 30				Day 90				Day 180				SEM^2^	*P*-Value					
	HA-GI	LA-GI	HA-GF	LA-GF	HA-GI	LA-GI	HA-GF	LA-GF	HA-GI	LA-GI	HA-GF	LA-GF	HA-GI	LA-GI	HA-GF	LA-GF		Trt	Day	Breed	Trt* Day	Trt* Breed	Day* Breed
Dry matter, %	31.07	32.50	32.02	31.21	30.92^a^	32.63^a^	27.63^b^	27.53^b^	29.53^a^	32.56^a^	25.66^b^	25.10^b^	29.08^a^	30.99^a^	22.18^b^	19.88^b^	1.33	<0.0001	<0.0001	0.022	**<0.0001**	0.572	0.075
Ammonia, mg/g	1.56	1.31	1.38	1.41	0.15	0.11	0.16	0.14	0.16	0.32	0.20	0.35	2.52^a^	1.52^b^	1.84^b^	1.88^b^	0.17	0.048	<0.0001	0.088	**0.010**	0.826	0.546
Phenol and Indole, µmol/g																							
Phenol	0.64	0.77	0.44	1.00	0.23	1.01	0.28	0.11	0.51^ab^	0.74^ab^	0.98^a^	0.26^b^	0.70	0.25	0.30	0.12	0.25	0.103	<0.001	0.371	**0.020**	0.455	0.227
4-Ethylphenol	3.32	2.64	2.58	2.91	1.95	2.81	1.90	0.90	4.16^a^	3.13^a^	3.09^a^	1.14^b^	2.44^a^	2.09^ab^	1.92^bc^	1.00^c^	0.54	0.029	<0.0001	0.995	**<0.001**	0.700	0.098
Indole	2.04	1.48	1.45	2.07	1.36^a^	1.68^a^	0.86^ab^	0.09^b^	2.40^a^	1.81^a^	1.97^a^	0.24^b^	1.71^a^	1.34^ab^	0.85^bc^	0.25^c^	0.31	<0.0001	<0.0001	0.004	**<0.0001**	0.972	0.992
SCFA^3^, µmol/g																							
Acetate	246.26	245.42^y^	237.74^x^	230.45^x^	385.87	364.23^y^	392.95^x^	403.01^x^	307.34	247.71^y^	286.07^x^	239.37^x^	245.78	218.27^y^	305.97^x^	290.67^x^	24.72	**0.009**	**<0.0001**	**0.023**	0.436	0.122	0.699
Propionate	139.14	163.45	144.23	130.87^‡^	191.2^b^	178.72^b^	200.66^b^	249.77^a,‡^	150.05^bc^	130.15^c^	183.54a^b^	161.41^a,‡^	124.26^b^	108.24^b^	172.37^a^	184.63^a,‡^	18.41	<0.0001	<0.0001	<0.0001	**<0.001**	**0.040**	0.657
Butyrate	41.39	57.08	44.25^x^	45.71^y^	58.34	59.29	67.84^x^	38.86^y^	59.03	41.45	47.86^x^	32.23^y^	42.52	49.23	62.99^x^	44.56^y^	6.33	**0.032**	**0.032**	0.757	0.167	0.205	0.873
Total SCFA	426.79^y^	465.95^y^	426.22^x^	407.03^x^	635.41^y^	602.24^y^	661.45^x^	691.65^x^	516.42^y^	419.31^y^	517.47^x^	433.01^x^	412.56^y^	375.73^y^	541.33^x^	519.87^x^	44.52	**<0.001**	**<0.0001**	**0.002**	0.094	0.053	0.833
BCFA^4^, µmol/g																							
Isobutyrate	5.99	5.70	5.16	5.73	7.79^a^	6.79^a^	7.11^ab^	3.85^b^	7.27^a^	4.80^a^	5.78^a^	2.36^b^	8.54	5.38	6.79	6.96	1.03	<0.0001	0.001	0.246	**0.006**	0.051	0.574
Isovalerate	9.20	7.73	8.20	7.94	10.85^a^	10.22^a^	9.37^a^	5.73^b^	10.18^a^	7.75^a^	8.16^a^	3.92^b^	11.76^a^	7.85^ab^	8.33^b^	5.37^b^	1.02	<0.0001	0.017	0.139	**0.015**	0.352	0.318
Valerate	3.67	1.06	1.20	0.72^‡^	5.62	5.51	3.28	5.29^‡^	4.04^b,**^	2.33^b,**^	2.67^b,**^	2.54^a,**,‡^	1.77^b,**^	2.50^b,**^	3.26^b,**^	11.33^a,**,‡^	3.34	0.001	<0.0001	0.001	**<0.0001**	**0.007**	**<0.001**
Total BCFA	18.86	14.49	14.56	14.39^‡^	24.26	22.52	19.75	14.87^‡^	21.49^**^	14.88^**^	16.60^**^	8.82^**,‡^	22.06^b,**^	15.73^b,**^	18.38^b,**^	23.65^a,**,‡^	4.23	0.486	<0.0001	0.001	**0.002**	**0.024**	**0.010**

^1^Values are on a dry matter basis. Trt * Day * Breed interaction was not significant (*P* > 0.05) for any treatment. HA-GI, high animal protein grain-inclusive; LA-GF, low animal protein grain-free; LA-GI, low animal protein grain-inclusive; HA-GF, high animal protein grain-free.

^2^Standard error of the mean.

^3^Short-chain fatty acids.

^4^Branched-chain fatty acids.

^a,b,c^Within a row, treatment means with different superscripts within each day differ (*P *< 0.05).

^x,y^Within a row, treatment means with different superscripts differ (*P *< 0.05).

^**^Within a row, Beagles’ means with different superscripts differ from mixed-breed hounds within each day (*P *< 0.05).

^‡^Within a row, Beagles’ means with different superscripts differ from mixed-breed hounds within each treatment (*P *< 0.05).

A treatment-by-breed interaction (*P* < 0.05) was detected for fecal acetate and total SCFA concentrations. Mixed-breed hounds generally had a greater SCFA concentration than Beagles (*P* < 0.05). For mixed-breed hounds, LA-GF had greater (*P* < 0.05) concentrations of total SCFA (640.77 µmol/g) and acetate (339.07 µmol/g) than HA-GI (497.65 and 284.35 µmol/g, respectively) and LA-GI (500.64 and 285.36 µmol/g, respectively). A treatment-by-day interaction (*P* < 0.05) was depicted for fecal propionate concentration. On day 30, LA-GF had a greater propionate concentration (293.95 µmol/g) than HA-GI (191.19 µmol/g), LA-GI (187.72 µmol/g), and HA-GF (223.86 µmol/g) (*P* < 0.05). Similar results were observed on day 180; there was a greater fecal propionate concentration in LA-GF (227.82 µmol/g) compared to HA-GI (137.94 µmol/g) and LA-GI (125.96 µmol/g) (*P* < 0.05). The main effects of treatment and day (*P* < 0.05) were noted for fecal butyrate concentration; HA-GF (54.55%) had a greater (*P* < 0.05) concentration than LA-GF (45.13%), whereas day 30 (54.97%) had a greater (*P* < 0.05) fecal butyrate concentration than day 90 (46.46%).

For total BCFA, there was interaction for treatment-by-day (*P* < 0.05). On day 180, there was greater (*P* < 0.05) fecal total BCFA in LA-GF (37.50 µmol/g) than in HA-GI (21.44 µmol/g) and LA-GI (20.94 µmol/g. Regarding the breed effect, mixed-breed hounds had a greater (*P* < 0.05) fecal BCFA than Beagles. Fecal scores differed between breeds (*P* < 0.05), where mixed-breed hounds had a greater fecal score of 3.6 than Beagles (3.1).

Fecal BAs were analyzed for primary (CA, CDCA) and secondary (DCA, LCA, UDCA) BAs. **Tables 7 and 8** show the mean BA concentrations for mixed-breed hounds and Beagles, respectively. For fecal BAs, a treatment-by-day-breed interaction was observed only for primary (*P *= 0.0086) and CA (*P *= 0.0094). On day 30, for all dogs, LA-GF had a greater (*P *< 0.05) CA concentration (10.7 µg/g) than HA-GI (1.1 µg/g), LA-GI (1.2 µg/g), and HA-GF (4.3 µg/g). Considering different time points within LA-GF, day 30 had a greater (*P *< 0.05) CA concentration than days 0, 90, and 180. Comparing breeds, mixed-breed hounds had a greater (*P *< 0.05) CA concentration than Beagles. For mixed-breed hounds, LA-GF had a greater (*P *< 0.05) CA concentration (7.0 µmol/g) than HA-GI (1.7 µmol/g) and LA-GI (1.4 µmol/g). Day 30 also had greater (*P* < 0.05) CA concentration than days 0, 90, and 180 in mixed-breed hounds.

**Table 7.** Fecal bile acid concentrations of mixed-breed hounds fed experimental diets containing different sources of dietary proteins and carbohydrates

Bile acid^1^, μg/g	Day 0				Day 30				Day 90				Day 180				SEM^2^	*P*-value				
	HA-GI	LA-GI	HA-GF	LA-GF	HA-GI	LA-GI	HA-GF	LA-GF	HA-GI	LA-GI	HA-GF	LA-GF	HA-GI	LA-GI	HA-GF	LA-GF		Trt	Day	Breed	Trt^*^ Day	Day^*^ Breed
Primary—total^3^	0.92	0.88	1.28	1.05	0.85	0.92	3.73	8.60	1.06	0.73	1.55	1.79	1.00	0.87	1.81	1.61	0.75	0.0223	0.0066	0.0164	**0.0007**	**<0.0001**
CA^3^	0.72	0.62	0.95	0.76	0.58	0.59	3.29	7.92	0.74	0.42	1.22	1.36	0.72	0.68	1.53	1.33	0.73	0.0235	0.0154	0.0165	**0.0011**	**<0.0001**
CDCA	0.20	0.25	0.33	0.29	0.27^y,†^	0.33^y,†^	0.44^†^	0.67^x,†^	0.31	0.31	0.33	0.43	0.27	0.19	0.27	0.28	0.05	0.1462	<0.0001	0.1247	**0.0015**	**0.0193**
Secondary—total	6.27^c,*^	5.90^c^	8.80^c,*^	5.07^c,**^	11.67^a.*^	10.51^a^	17.93^a,*^	8.05^a.**^	11.53^ab.*^	8.76^ab^	9.20^ab.*^	4.31^ab,**^	10.81^bc,*^	7.26^bc^	6.69^bc,*^	4.35^bc,**^	2.31	**0.0018**	**<0.0001**	0.2611	0.1946	0.8013
DCA	4.93^c,*^	4.40^c^	7.11^c,*^	3.68^c,**^	10.42^a,*^	9.01^a^	16.85^a,*^	7.23^a,**^	9.47^b,*^	6.86^b^	7.80^b,*^	3.39^b,**^	9.36^bc,*^	6.15^bc^	5.69^bc,*^	3.70^bc,**^	2.23	**0.0027**	**<0.0001**	0.3039	0.2244	0.7676
LCA	1.03	1.10	1.35	1.04	0.90^x^	0.97^x^	0.78	0.46^y^	1.72^x^	1.57^xy^	1.07^y^	0.63^z^	1.14^x^	0.81^x^	0.72^y^	0.40^y^	0.15	<0.0001	<0.0001	0.0218	**0.0002**	0.8057
UDCA	0.31^y^	0.40^x^	0.34	0.35^x^	0.35^y^	0.53^x^	0.30^y^	0.37^y^	0.34	0.33	0.33	0.30	0.31	0.30	0.27	0.26	0.05	0.008	<0.0001	0.4401	**0.0342**	0.5970

^1^Values are on a dry matter basis. Trt * Breed was not significant (*P* > 0.05) for all variables. HA-GI, high animal protein grain-inclusive; LA-GF, low animal protein grain-free; LA-GI, low animal protein grain-inclusive; HA-GF, high animal protein grain-free; CA, cholic acid; CDCA, chenodeoxycholic acid; DCA, deoxycholic acid; LCA, lithocholic acid; UDCA, ursodeoxycholic acid.

^2^Standard error of the mean.

^3^Trt * Day * Breed interaction was not significant (*P* > 0.05) for most variables, except for total primary (*P* = 0.0086) and CA (*P* = 0.0094), where LA-GF on day 30 for mixed-breed hounds was significantly greater than all other timepoints, diets, and breeds.

^x,z^Within a row, treatment means with different superscripts within each day differ (*P *< 0.05).

^*^Within a row, treatment means with different superscripts differ (*P *< 0.05).

^a,b,c^Within a row, day means with different superscripts differ (*P *< 0.05).

^†^Within a row, mixed-breed hounds’ means with different superscripts differ from Beagles within each day (*P *< 0.05).

**Table 8.** Fecal bile acid concentration of Beagles fed experimental diets containing different sources of dietary proteins and carbohydrates

Bile acid^1^, μg/g	Day 0				Day 30				Day 90				Day 180				SEM^2^	*P*-value				
	HA-GI	LA-GI	HA-GF	LA-GF	HA-GI	LA-GI	HA-GF	LA-GF	HA-GI	LA-GI	HA-GF	LA-GF	HA-GI	LA-GI	HA-GF	LA-GF		Trt	Day	Breed	Trt^*^ Day	Day^*^ Breed
Primary—total^3^	1.13	1.70	1.24	1.03	0.69	0.73	0.48	1.14	2.05	0.77	0.90	1.76	0.90	0.42	0.50	0.71	1.13	0.0223	0.0066	0.0164	**0.0007**	**<0.0001**
CA^3^	0.89	1.26	0.90	0.82	0.34	0.43	0.19	0.79	1.72	0.49	0.59	1.37	0.67	0.22	0.38	0.52	0.89	0.0235	0.0154	0.0165	**0.0011**	**<0.0001**
CDCA	0.24	0.44	0.34	0.21	0.34^y^	0.30^y^	0.29	0.35^x^	0.33	0.29	0.32	0.39	0.23	0.21	0.12	0.19	0.24	0.1462	<0.0001	0.1247	**0.0015**	**0.0193**
Secondary—total	5.04^c,*^	3.90^c^	5.05^c,*^	5.01^c,**^	16.38^a,*^	8.90^a^	12.38^a,*^	4.33^a,**^	10.93^ab,*^	8.38^ab^	9.84^ab,*^	5.38^ab.**^	9.49^bv,*^	5.16^bc^	5.47^bc,*^	3.61^bc,**^	5.04	**0.0018**	**<0.0001**	0.2611	0.1946	0.8013
DCA	3.81^c,*^	2.51^c,^	3.69^c,*^	3.73^c,**^	15.08^a,*^	7.66^a^	11.41^a,*^	3.55^a,**^	9.20^b,*^	6.86^b^	8.49^b,*^	4.45^b,**^	8.18^bc,*^	4.09^bc^	4.68^bc,*^	3.01^bc,**^	3.81	**0.0027**	**<0.0001**	0.3039	0.2244	0.7676
LCA	0.92	0.86	0.93	0.94	0.89^x^	0.73^x^	0.63	0.41^y^	1.39^x^	1.22^xy^	1.03^y^	0.62^z^	0.99	0.79	0.53	0.34	0.92	<0.0001	<0.0001	0.0218	**0.0002**	0.8057
UDCA	0.31^y^	0.54^x^	0.43	0.34^x^	0.40^y^	0.51^x^	0.33^y^	0.37^y^	0.34	0.31	0.31	0.31	0.32	0.27	0.26	0.26	0.31	0.008	<0.0001	0.4401	**0.0342**	0.5970

^1^Values are on a dry matter basis. Trt * Breed was not significant (*P* > 0.05) for all variables. HA-GI, high animal protein grain-inclusive; LA-GF, low animal protein grain-free; LA-GI, low animal protein grain-inclusive; HA-GF, high animal protein grain-free; CA, cholic acid; CDCA, chenodeoxycholic acid; DCA, deoxycholic acid; LCA, lithocholic acid; UDCA, ursodeoxycholic acid.

^2^Standard error of the mean.

^3^Trt * Day * Breed interaction was not significant (*P* > 0.05) for most variables, except for total primary (*P* = 0.0086) and CA (*P* = 0.0094), where LA-GF on day 30 for mixed-breed hounds was significantly greater than all other timepoints, diets, and breeds.

^x,z^Within a row, treatment means with different superscripts within each day differ (*P *< 0.05).

^*^Within a row, treatment means with different superscripts differ (*P *< 0.05).

^a,b,c^Within a row, day means with different superscripts differ (*P *< 0.05).

Interactions (*P *< 0.05) between treatment-by-day and day-by-breed were observed for fecal CDCA concentration. On day 30 for all dogs, LA-GF had a greater (*P *< 0.05) CDCA concentration (1.3 µg/g) than HA-GI (0.8 µg/g) and LA-GI (0.8 µg/g). Comparing different time points within LA-GF, day 30 had a greater (*P* < 0.05) CDCA concentration than day 0 (0.6 µg/g) and day 180 (0.6 µg/g). For Beagles, day 180 had a lower (*P* < 0.05) CDCA concentration compared with days 0, 30, and 90. Similarly, day 180 had a lower CDCA concentration for mixed-breed hounds than days 0 and 30 (*P* < 0.05).

A treatment effect (*P* < 0.05) was observed for fecal DCA concentration, with LA-GF having a lower DCA concentration (10.4 µg/g) compared with HA-GI (22.4 µg/g) and HA-GF (20.9 µg/g) (*P* < 0.05). An interaction (*P *< 0.05) between treatment and day was noted for LCA and UDCA.

On days 90 and 180, there were significantly greater (*P* < 0.05) concentrations of LCA observed in HA-GI (4.1 and 2.8 µg/g, respectively) than in LA-GF (1.7 and 1.0 µg/g, respectively). Within LA-GF, day 0 had a greater (*P* < 0.05) LCA concentration compared with days 30 and 180 but not day 90. Within LA-GI, day 30 had a greater (*P* < 0.05) UDCA concentration compared with days 90 and 180.

### Fecal microbiota

After prevalence filtering, 157 taxa were identified in dogs fed diets containing different protein and carbohydrate sources. Firmicutes and Bacteroidota were the most common phyla in most samples. Fusobacteriota also had a high abundance in a few dogs (**Supplementary Figure S1**). Fecal samples from dogs fed LA-GF had a greater proportion of Firmicutes and a lower proportion of Fusobacteriota than the other treatments. Mixed-breed hounds generally had more Firmicutes and less Fusobacteriota than Beagles (**Supplementary Figure S2**).

For family composition, fecal samples of LA-GF had a greater proportion of Lactobacillaceae, Selenomonadaceae, and Streptococcaceae than the other treatments ­(**Supplementary Figure S3**). There was also an increase in Veillonellaceae for LA-GF in contrast with other treatments (**Supplementary Figure S4**). Similarly, genus composition varied among samples, with Ligilactobacillus, Prevotella 9, Peptoclostridium, Bacteroides, and Fusobacterium being common in most fecal samples. At the same time, *Megamonas* was frequently observed in fecal samples of dogs fed LA-GF (**Supplementary Figure S5**). The relative abundance of *Megamonas* was prevalent in LA-GF from day 30 onward (**Supplementary Figure S6**). The relative abundance of *Ligilactobacillus* and *Streptococcus* also increased over time in fecal samples of dogs fed LA-GF.

**Figure 1.**
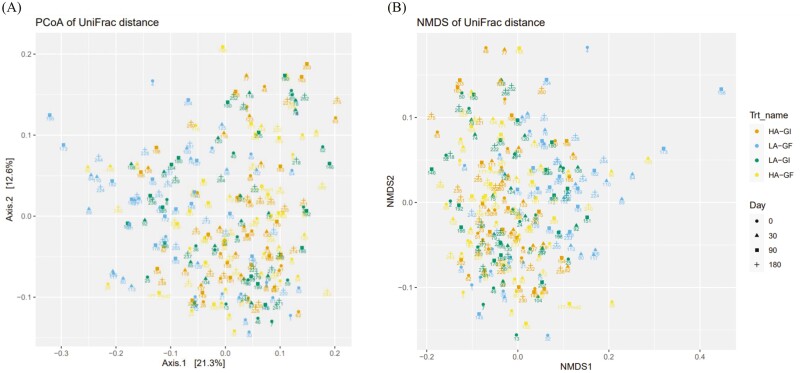
Principal coordinated plot of unweighted UniFrac distances (**A**) and nonmetric multidimensional scaling (NMDS) of UniFrac distances (**B**) of fecal microbial communities of dogs fed experimental diets containing different sources of dietary proteins and carbohydrates.

**Figure 2.**
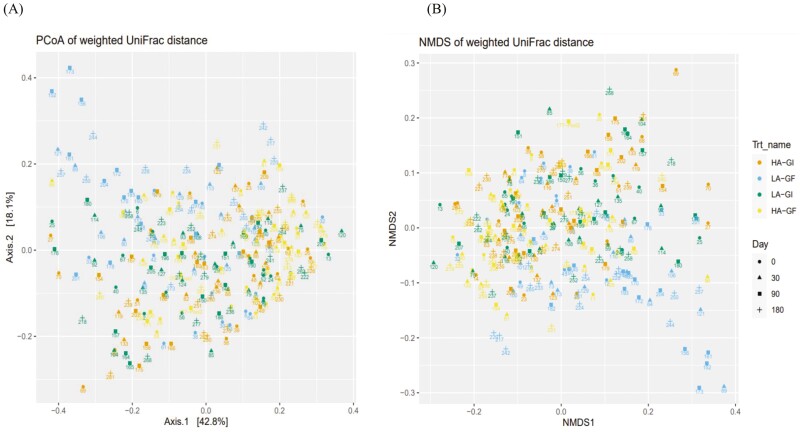
Principal coordinated plot of weighted UniFrac distances (**A**) and nonmetric multidimensional scaling (NMDS) of UniFrac distances (**B**) of fecal microbial communities of dogs fed experimental diets containing different sources of dietary proteins and carbohydrates.

**Figure 3.**
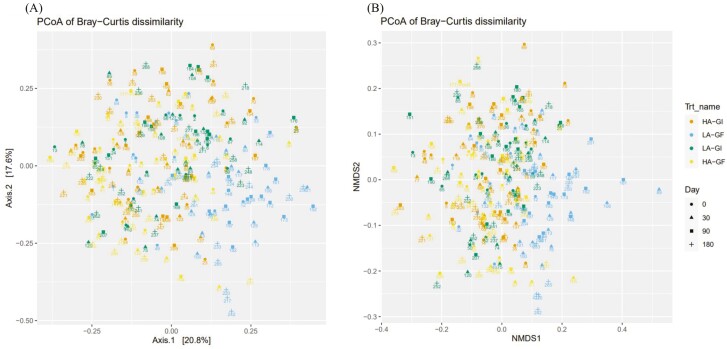
Principal coordinated plot of Bray–Curtis dissimilarity (**A**) and nonmetric multidimensional scaling (NMDS) of Bray–Curtis dissimilarity (**B**) of fecal microbial communities of dogs fed experimental diets containing different sources of dietary proteins and carbohydrates.

**Figure 4.**
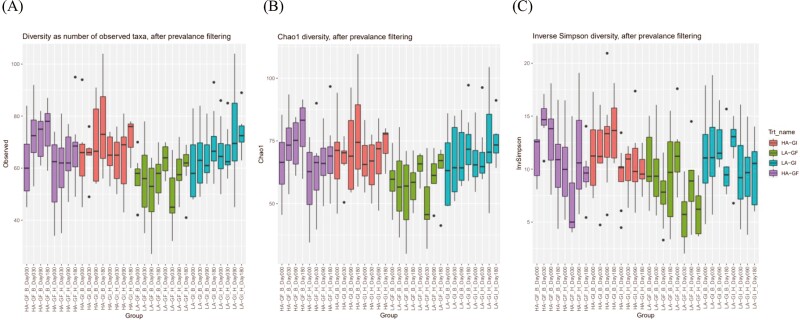
Fecal microbial α-diversity as (**A**) observed taxa, (**B**) Chao 1, and (**C**) Inversed Simpson diversity of dogs fed experimental diets containing different sources of dietary proteins and carbohydrates after prevalence filtering.

Principal coordinates analysis (**PCoA**) and NMDS were used to visualize the distance matrices in three dimensions (**Figs. 1 to 3**). Fecal samples from dogs on LA-GF were significantly different from the other treatments from day 30 onward using Bray–Curtis dissimilarity, unweighted UniFrac distances, and weighted UniFrac distances (*P* < 0.05). The α-diversity (measured as observed taxa, Chao1, and Inversed Simpson analyses) is shown in **Fig. 4**. No significant difference in α-diversity was observed at day 0 (*P *> 0.05). Fecal samples from LA-GF from day 30 onward had lower diversity than the other three treatments for all three types of analyses.

The differential abundance of microbial communities is shown in **Table 9**. The difference is characterized as a log 3-fold change, with a *P*-value and FDR cutoff of 0.05. Treatment LA-GF was the most different from the others with a greater abundance of *Megasphaera elsdenii* and *Bifidobacterium* sp. than the high animal protein groups, HA-GI and HA-GF (*P* < 0.05). There was also a lower (*P* < 0.05) abundance of *Lachnospiraceae sp.* and a greater abundance of *Collinsella aerofaciens* in dogs fed LA-GF compared with HA-GI. *Peptococcus* sp. was less abundant (*P* < 0.05) in LA-GF than in LA-GI. At day 0, there was no effect of breed in any treatment groups. On day 180, only dogs fed LA-GF showed a breed difference as mixed-breed hounds had more *Megasphaera elsdenii* and less *Bifidobacterium* sp. compared with Beagles (*P* < 0.05).

**Table 9.** Differential abundance of microbial communities in canines fed experimental diets containing different sources of dietary proteins and carbohydrates at day 180 with a minimum of 3-log-fold change

Phylum	Family	Genus	Species	Log fold change
LA-GF vs. HA-GI				
Firmicutes	Lachnospiraceae	NA	Lachnospiraceae sp.	−3.3
Firmicutes	Veillonellaceae	Megasphaera	elsdenii	3.9
Actinobacteriota	Coriobacteriaceae	Collinsella	aerofaciens	3.8
Actinobacteriota	Bifidobacteriaceae	Bifidobacterium	Bifidobacterium sp.	3.8
LA-GI vs. LA-GF				
Firmicutes	Peptococcaceae	Peptococcus	Peptococcus sp.	3.1
HA-GF vs. LA-GF				
Firmicutes	Veillonellaceae	Megasphaera	elsdenii	−3.1
Actinobacteriota	Bifidobacteriaceae	Bifidobacterium	Bifidobacterium sp.	−3.4
Hound vs. Beagle in LA-GF				
Firmicutes	Veillonellaceae	Megasphaera	elsdenii	4.2
Actinobacteriota	Bifidobacteriaceae	Bifidobacterium	Bifidobacterium sp.	−3.0

HA-GI, high animal protein diet; LA-GF, low animal protein grain-free diet; LA-GI, low animal protein diet; HA-GF, high animal protein grain-free diet.

**Figure 5.**
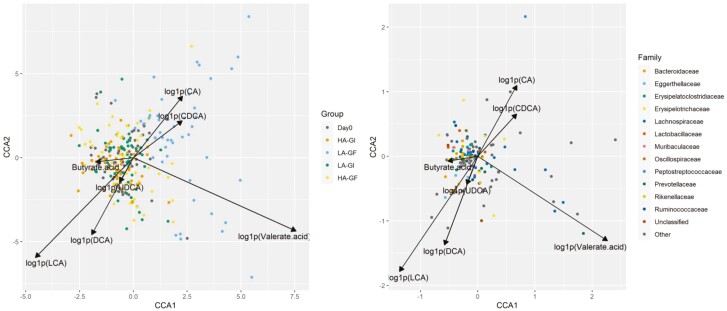
Canonical correspondence analysis of taxa abundance.

Canonical correspondence analysis of taxa abundance constrained by fecal metabolites is shown in **Fig. 5**. The differential abundance was filtered by a detection rate of no fewer than 10% of the samples. Treatment LA-GF was positively correlated with fecal CA, CDCA, and valerate concentration. Fecal valerate concentration was positively related to *Megasphaera elsdenii*, *Collinsella aerofaciens*, and *Bifidobacterium sp* (*P* < 0.05).

## Discussion

These diets were formulated to be the same in macronutrient concentration. Upon testing, macronutrients had similar concentrations. Overall, the data supports the hypothesis that high and low animal-sourced protein diets, varying in ­carbohydrate sources are well tolerated and digested by the current study’s cohort of dogs. Additionally, the varying carbohydrate sources and fiber type and amount do impact the fermentation end products. Since no difference was seen in food intake among treatments, any treatment effect observed in different parameters in the current study is likely a result of the variance in the amount of animal protein or ingredients. The differences in fecal output on a DM basis according to breed could be due to the differences in the digestibility of macronutrients and fecal moisture content. Similar findings in fecal output were shown in a previous study which included dogs of different sizes with BW ranging from 4 to 50 kg (Weber et al., 2003). The high fecal output in dogs fed the LA-GF diet aligns with similar findings from several studies that have observed increased fecal output with plant-based diets compared with animal-based diets and grain-free diets compared with grain-inclusive diets (Bednar et al., 2000; Hill et al., 2001; Pezzali and Aldrich, 2019; Reilly et al., 2021b). The fecal output difference from Hill et al. (2001) could be due to the greater fiber content in plant-based diets binding more water due to increased bulk volume (Dai and Chau, 2017). Both studies from Pezzali and Aldrich (2019) and Reilly et al. (2021b) used Beagles fed either grain-free diets or grain-inclusive diets; the two types of diets were similar in TDF content, but the grain-free diets had greater soluble fiber content compared to the grain-inclusive diets. Therefore, a greater fermentation rate with the soluble fiber from pulse ingredients could contribute to the difference in the fecal output (Bednar et al., 2001). However, the chemical composition would unlikely cause the difference in fecal output in the current study as all diets were comparable in TDF and soluble fiber contents. The lower fecal output in dogs fed HA-GF could be reflected in the consistently greater ATTD of all macronutrients of HA-GF in the present study.

All diets could be considered well-digested by both Beagles and mixed-breed hounds with greater than 80% ATTD. There were greater ATTD for DM, OM, AHF, CP, and TDF in mixed-breed hounds than in Beagles. This corresponds with previous studies showing large breed dogs had greater digestibility and fermentative activities than small-breed dogs (Weber et al., 2003, 2004). The studies from Weber et al. (2003, 2004) included four dog breeds: 6 miniature Poodles (BW 3.5 ± 0.7 kg), 6 medium Schnauzers (BW 12.6 ± 0.9 kg), 6 Giant Schnauzers (BW 23.3 ± 1.3 kg), and 6 Great Danes (BW 46.3 ± 1.0 kg). They observed greater ATTD for DM, OM, and CP in larger-sized breeds when fed the same kibble diet. There was also greater fecal moisture, lactate, and total SCFA concentration in larger breeds, indicating greater fermentative activity. However, another study by Meyer et al. (1999) only observed little difference in ATTD from body size, which included ten breeds with BW ranging from 4.2 to 52.5 kg.

Several canine studies have investigated the effects of plant protein in contrast with animal protein on ATTD (Bednar et al., 2000; Hill et al., 2001; Vanelli et al., 2021). In agreement with previous literature, in the current study, Beagles fed LA-GI had lower OM and AHF digestibility than Beagles fed HA-GI. Still, these differences were not present in mixed-breed hounds. For ATTD of TDF, there was no difference among treatments in mixed-breed hounds. However, in Beagles, HA-GF resulted in the greatest ATTD of TDF, while the other three treatments did not differ. This was an intriguing finding because the TDF content and soluble-to-insoluble fiber ratio were similar among all diets. Other studies also found increases in TDF digestibility when dogs were fed grain-free diets, but this was due to the greater soluble fiber content in the diets (Carciofi et al., 2008; Pezzali and Aldrich, 2019). The digestible energy was lowest in Beagles fed LA-GI, which corresponded to the lower macronutrient ATTD. Additionally, the digestibility of diets has been related to a greater gelatinization, obtained during the extrusion process, in pulses that are typically greater inclusions in grain-free diets (Monti et al., 2016; Alvarengo et al., 2021).

Fresh fecal DM content was lower in both grain-free treatment groups (LA-GF and HA-GF) than in grain-inclusive treatments (LA-GI and HA-GI). This finding is in accordance with other studies testing diets, including legumes (Carciofi et al., 2008; Pezzali and Aldrich, 2019). Dogs fed the diets containing peas, lentils, and cassava flour had lower fecal DM content than those fed the brewer’s rice diet (Carciofi et al., 2008). Similarly, Pezzali and Aldrich (2019) found a lower fecal DM in Beagles fed the grain-free diet, which contained potato, peas, and tapioca starch, compared with those fed the ancient grain diet, which included spelt, millet, and sorghum. In the current study, a breed effect was also seen, as mixed-breed hounds had lower fecal DM and greater fecal scores compared with Beagles.

Weber et al. (2004) observed a positive correlation between fecal moisture and body size/weight, hypothesizing that lower fecal DM or greater moisture content could respond to increased hindgut fermentation in larger dogs. Thus, greater fecal concentrations of fermentation end products, including total SCFA, total BCFA, and ammonia in mixed-breed hounds compared with Beagles were expected because larger dogs have longer colonic transit time, which favors the fermentation (Macfarlane and Macfarlane, 2003). Weber et al. (2004) also found a positive correlation between fecal total SCFA concentration and body size/weight. The high organic acid concentration in the lumen could also lead to looser stools, which could be related to lower fecal DM in mixed-breed hounds (Weber et al., 2017). In contrast, the lower fecal indole concentration in mixed-breed hounds compared with Beagles could be related to less protein entering the large intestine because of the greater protein digestibility observed in larger breed dogs (Weber et al., 2003).

Regarding dietary treatments, dogs fed HA-GI had the greatest fecal ammonia and indole concentrations, while dogs fed LA-GF had the lowest. This corroborates with a study from Nery et al. (2012), reporting lower fecal concentrations of protein fermentative end products in a plant protein-based diet (wheat gluten) compared with a poultry meal diet in 27 dogs differing in body size. It was proposed that the lower fecal protein metabolites were driven by the greater protein digestibility of the plant-based diet and less protein reaching the colon. The grain-free diets resulted in greater fecal total SCFA, acetate, and propionate concentrations than grain-inclusive diets. The difference could be due to pea fiber inclusion in grain-free diets being available for fermentation. The finding is consistent with a study by (Reilly et al., 2021b), which demonstrated greater fecal total SCFA concentrations in plant-based diets made with legumes when compared with a poultry-based diet in dogs.

BA metabolism is closely linked to the taurine homeostasis (Czuba and Vessey, 1981); thus, fecal BA could indicate a taurine loss. It has been hypothesized that high levels of fermentable fibers could lead to losses of taurine through the BA cycle (Mansilla et al., 2019). However, in 2022, preliminary data presented at the American Academy of Veterinary Nutrition symposium from a study with mixed-breed hounds and Beagles fed diets with or without pulse ingredients and those of high and low animal protein content suggested that breed may affect whole blood taurine concentrations, as well as dogs fed high-animal grain-free diets had lower plasma taurine compared to the other diets; however, plasma taurine concentrations increased throughout the study compared to baseline (Streeter et al., 2022). Furthermore, Torres et al. (2022), observed that whole blood and plasma taurine may not be a sensitive enough biomarker to assess deficiencies in dogs fed diets lower in sulfur-containing amino acids; and that dietary taurine may not correlate with circulating taurine (Freeman et al., 2001). In the current study, mixed-breed hounds fed grain-free diets had greater fecal primary BA concentration but lower secondary BA concentration than grain-inclusive diets. A 4-wk study from Pezzali et al. (2020) included 12 adult neutered Beagles fed either a grain-based diet containing sorghum, millet, and spelt or a grain-free diet containing potatoes, peas, and tapioca starch in a completely randomized design. They also found similar results as the proportion of primary BA in feces was greater in dogs fed the grain-free diet compared with the grain-based diet. Another 90-d study from Reilly et al. (2021a), including 12 intact adult Beagles, had consistent findings and showed greater fecal primary BA concentration and lower secondary BA content in dogs fed a green lentil diet compared with a poultry-based diet. A 7-d study by Quilliam et al. (2021) which included eight adult neutered Beagles, demonstrated that the inclusion of pulses did not increase BA losses in feces or induce a decline in fasted plasma taurine level. However, the study from Quilliam et al. (2021) was relatively short compared with the current study, and the studies from Pezzali et al. (2020) and Reilly et al. (2021a), which could be a factor in the conflicting results in fecal BA concentrations. While the ideal ratio of primary to secondary BAs for the overall health of the animal is not fully understood, greater primary BA concentrations observed could be a result of gut microbiota deconjugation (Pezzali et al., 2020). This could suggest that the bacterial enzyme 7-alpha dehydroxylase, which is responsible for the dehydroxylation of primary BAs to secondary in the colon, could be inhibited. This is noteworthy as a shift in alpha diversity was observed in the LA-GF diet, even though there was no statistically significant difference in soluble fiber among diets, which has been previously hypothesized (Pezzali et al., 2020) to be a factor in the observed shift. Differences were observed in the BAs between treatments; yet in this larger portion of this study, plasma taurine was not negatively affected (Streeter et al., 2022); thus, future studies should investigate this further to understand the most appropriate proportion of primary and secondary BAs.

The most abundant fecal microbiota from the current study was Firmicutes and Bacteroidota. These two phyla are commonly represented in abundance in canine fecal samples according to previous studies (Kerr et al., 2013; Reddy et al., 2019; Sandri et al., 2019; Reilly et al., 2021b). It was observed that mixed-breed hounds had more Firmicutes and fewer Fusobacteriota than Beagles. Reddy et al. (2019) also saw this breed difference in a study that found a lower abundance of Firmicutes and a greater abundance of Fusobacteriota in dogs weighing between 3.8 and 7.6 kg when fed the same diet for 3 wk. Considering the treatment effect, dogs fed LA-GF had the most different composition with greater Selenomonadaceae, Veillonellaceae, Lactobacillaceae, *Streptococcus*, *Ligilactobacillus*, *Megamonas*, *Collinsella aerofaciens*, and *Bifidobacterium* sp. than the other groups. Selenomonadaceae have previously been related to an increased level of acetate concentration and decreased propionate (Kim et al., 2021). Selenomonadaceae and Veillonellaceae are considered amino acid fermenting bacteria (Dai et al., 2011). Therefore, it was expected that the abundance of Veillonellaceae had a positive correlation with fecal valerate concentration in the current study. Reilly et al. (2021b) also found a greater abundance of Veillonellaceae in dogs fed a pulse-based diet when compared to the control poultry diet. Within the Veillonellaceae family, *Megasphaera elsdenii* was specifically greater in differential abundance in LA-GF than the high animal protein groups (HA-GI and HA-GF). A previous in vitro model for humans also showed that *Megasphaera elsdenii* increased with added pulses (Rose et al., 2021).

On the other hand, many strains in the Lactobacillaceae family, *Bifidobacterium,* and *Streptococcus* are commonly viewed as beneficial to host health and are used in probiotics (Baillon et al., 2004; Garcia-Mazcorro et al., 2011; Ciaravolo et al., 2021. A specific strain of *Lactobacillus acidophilus,* when incorporated into dry dog food, demonstrated probiotic functions in adult dogs (Baillon et al., 2004). One study by Jimenez-Trigos et al. (2022) evaluated the potential of *Ligilactobacillus salivarius* being a probiotic in dogs and found functional probiotic attributes in an in vitro analysis. The increase in *Megamonas* was also depicted in previous research with dogs fed pulse-containing diets compared with grain-inclusive diets (Sandri et al., 2019; Reilly et al., 2021b). Studies have also noted an increase in *Megamonas* in dogs fed diets with inulin or fructooligosaccharides (Beloshapka et al., 2013; Sandri et al., 2019). The finding of a greater abundance of *Megamonas* from dogs fed LA-GF in the current study is consistent with previous research and could be a sign of adaptation to the grain-free diet because *Megamonas* produces enzymes to degrade galactooligosaccharides which are high in pulses (Polansky et al., 2015). Both PCoA and NMDS showed that LA-GF was separated from the other three treatments. Fecal samples from LA-GF had lower α-diversity compared with the others. However, no differences in fecal microbiota diversity and richness were observed in dogs with congestive heart failure (Seo et al., 2020; Araki et al., 2022). Moreover, despite the lower diversity observed in the LA-GF group, all dogs remained healthy throughout the study. In addition, the combination of low animal protein and legumes in the diet produced a beneficial shift in fecal microbiota, while the other treatments did not differ significantly.

## Conclusion

All experimental diets regardless of the protein or carbohydrate source were well-digested by both Beagles and mixed-breed hounds. Breed difference was seen in digestibility, fermentative end products, and fecal microbiota. Mixed-breed hounds had an overall greater macronutrient ATTD, greater fecal total SCFA concentration, greater fecal BA concentration, and a greater proportion of Firmicutes in fecal samples. The LA-GF diet resulted in lower fecal ammonia, indole, and secondary BA concentrations but greater total SCFA, acetate, propionate, and primary BA concentrations. LA-GF also shifted the fecal microbiota with more abundant Selenomonadaceae, Veillonellaceae, Lactobacillaceae, *Streptococcus*, *Ligilactobacillus*, *Megamonas*, *Collinsella aerofaciens*, and *Bifidobacterium* sp., and while a reduction in diversity was noted, all dogs remained healthy. Future studies should investigate shifts in microbiota and the overall health of the animals fed different diets.

## Supplementary Material

skad268_suppl_Supplementary_Figures

## Abbreviations

AAFCO: Association of American Feed Control Officials
AHF: acid-hydrolyzed fat
ASV: amplicon sequence variants
ATTD: apparent total tract digestibility
BA: bile acid
BCFA: branched-chain fatty acid
BCS: body condition score
BW: body weight
CA: cholic acid
CDCA: chenodeoxycholic acid
CP: crude protein
DCA: deoxycholic acid
DCM: dilated cardiomyopathy
DM: dry matter
FDR: false discovery rate
GE: gross energy
LCA: lithocholic acid
NMDS: non-metric multidimensional scaling
OM: organic matter
PCoA: principal coordinates analysis
SCFA: short-chain fatty acid
TDF: total dietary fiber
UDCA: ursodeoxycholic acid
