# Supplementary material for: The impact of protein source and grain inclusion on digestibility, fecal metabolites, and fecal microbiome in adult canines
Source: J Anim Sci. 2023 Aug 9;101:skad268. doi: 10.1093/jas/skad268 (PMC10464515; doi:10.1093/jas/skad268)
Supplement: skad268_suppl_Supplementary_Figures [file skad268_suppl_supplementary_figures.docx]

**Supplemental Figure 1.** Phylum composition of canine fecal samples displayed by treatment.


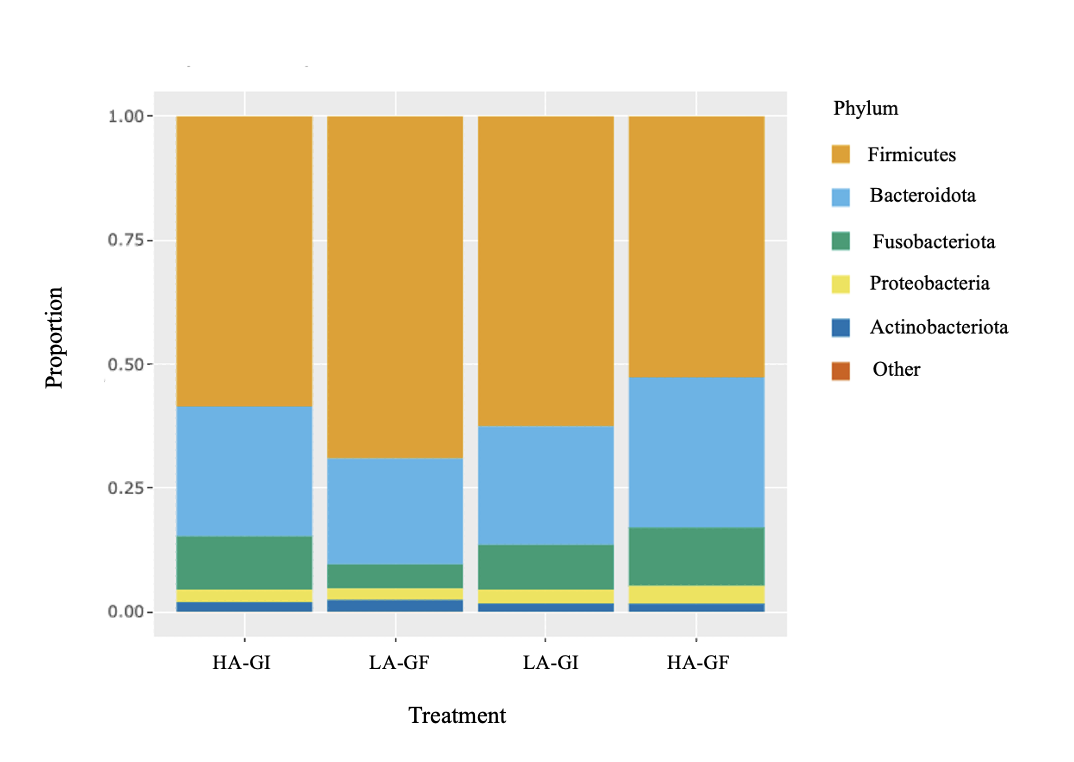


HA-GI: high animal protein grain-inclusive diet, LA-GF: low animal protein grain-free diet, LA-GI: low animal protein grain-inclusive diet, HA-GF: high animal protein grain-free diet.

**Supplemental Figure 2.** Phylum composition of canine fecal samples displayed by breed within treatment.


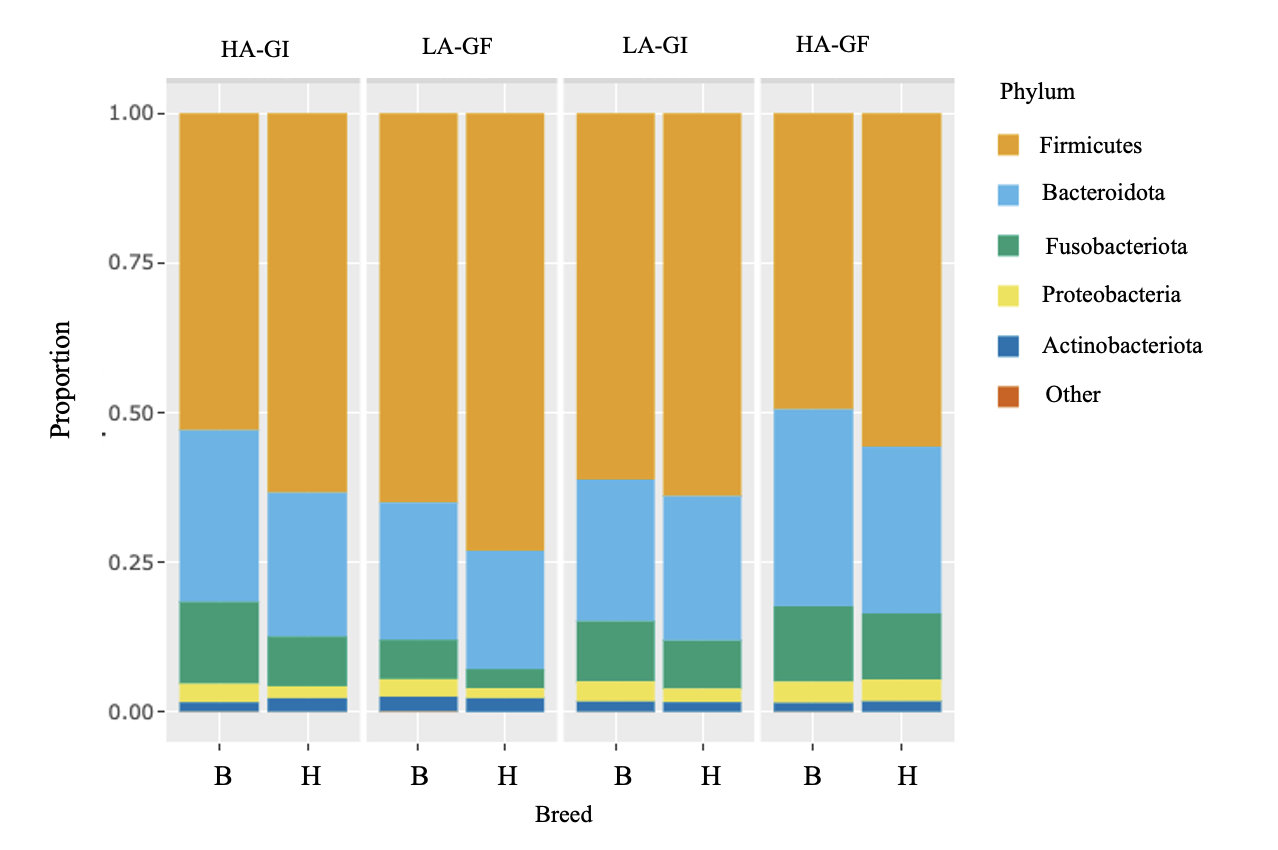


HA-GI: high animal protein grain-inclusive diet, LA-GF: low animal protein grain-free diet, LA-GI: low animal protein grain-inclusive diet, HA-GF: high animal protein grain-free diet.

B: beagle, H: mixed-breed hound.

**Supplemental Figure 3.** Family composition of canine fecal samples displayed by treatment.


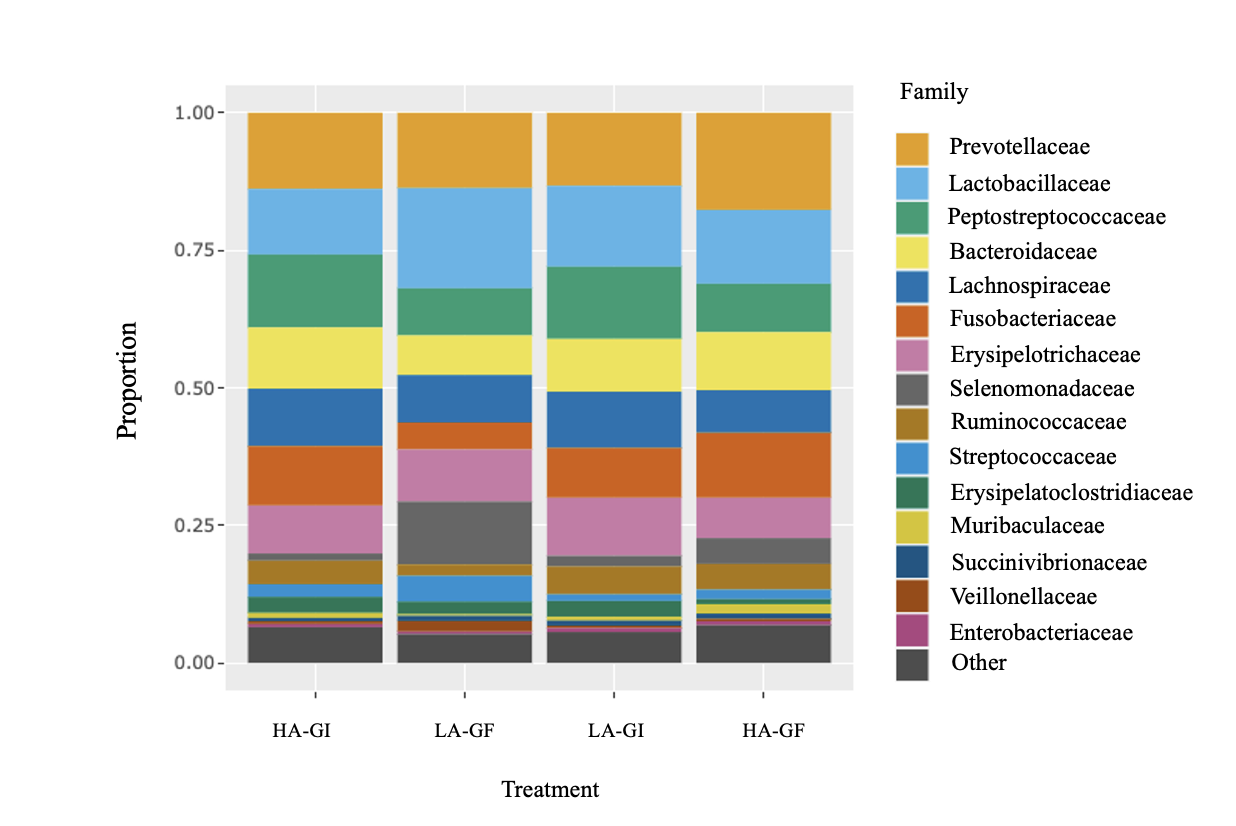


HA-GI: high animal protein grain-inclusive diet, LA-GF: low animal protein grain-free diet, LA-GI: low animal protein grain-inclusive diet, HA-GF: high animal protein grain-free diet.

**Supplemental Figure 4.** Family composition of canine fecal samples displayed by breed within treatment.


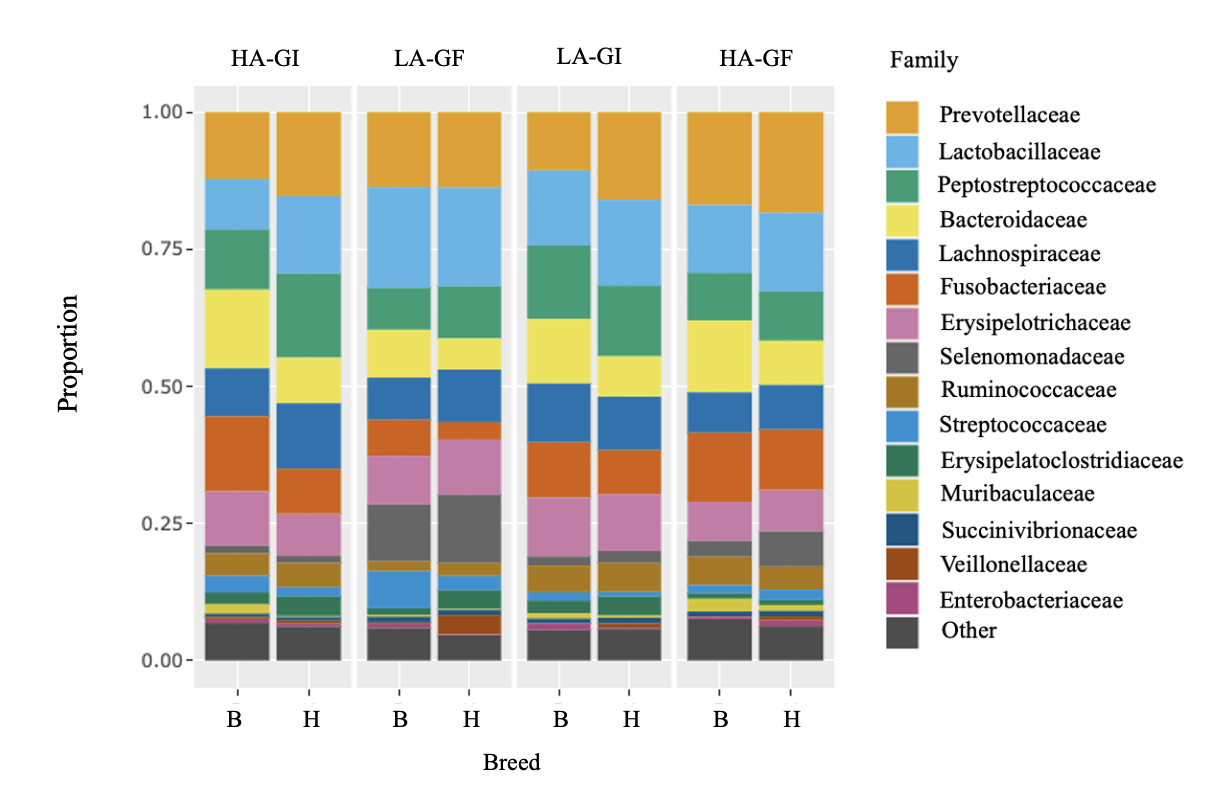


HA-GI: high animal protein grain-inclusive diet, LA-GF: low animal protein grain-free diet, LA-GI: low animal protein grain-inclusive diet, HA-GF: high animal protein grain-free diet.

B: beagle, H: mixed-breed hound.

**Supplemental Figure 5.** Genera composition of canine fecal samples displayed by treatment.


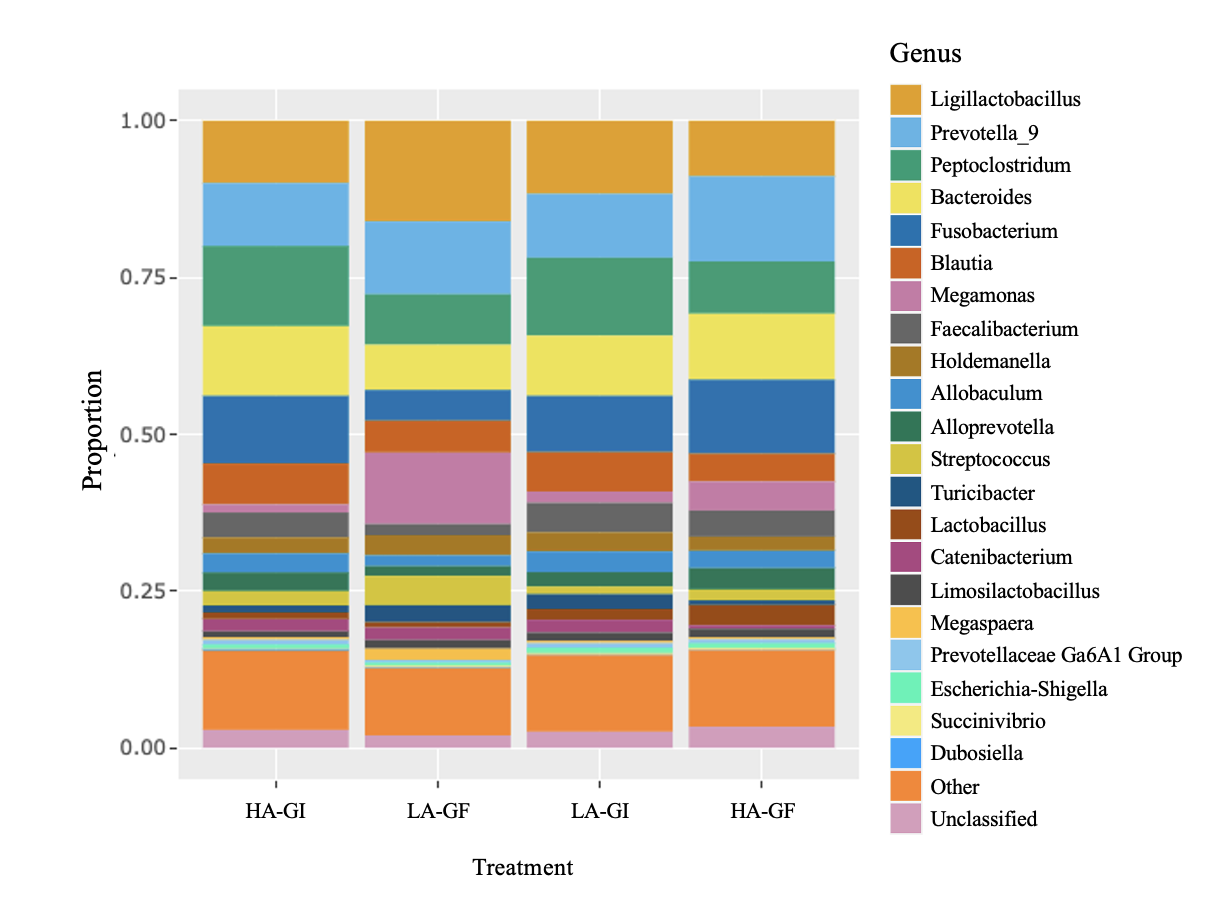


HA-GI: high animal protein grain-inclusive diet, LA-GF: low animal protein grain-free diet, LA-GI: low animal protein grain-inclusive diet, HA-GF: high animal protein grain-free diet.

**Supplemental Figure 6.** Genera composition of canine fecal samples displayed by treatment

.
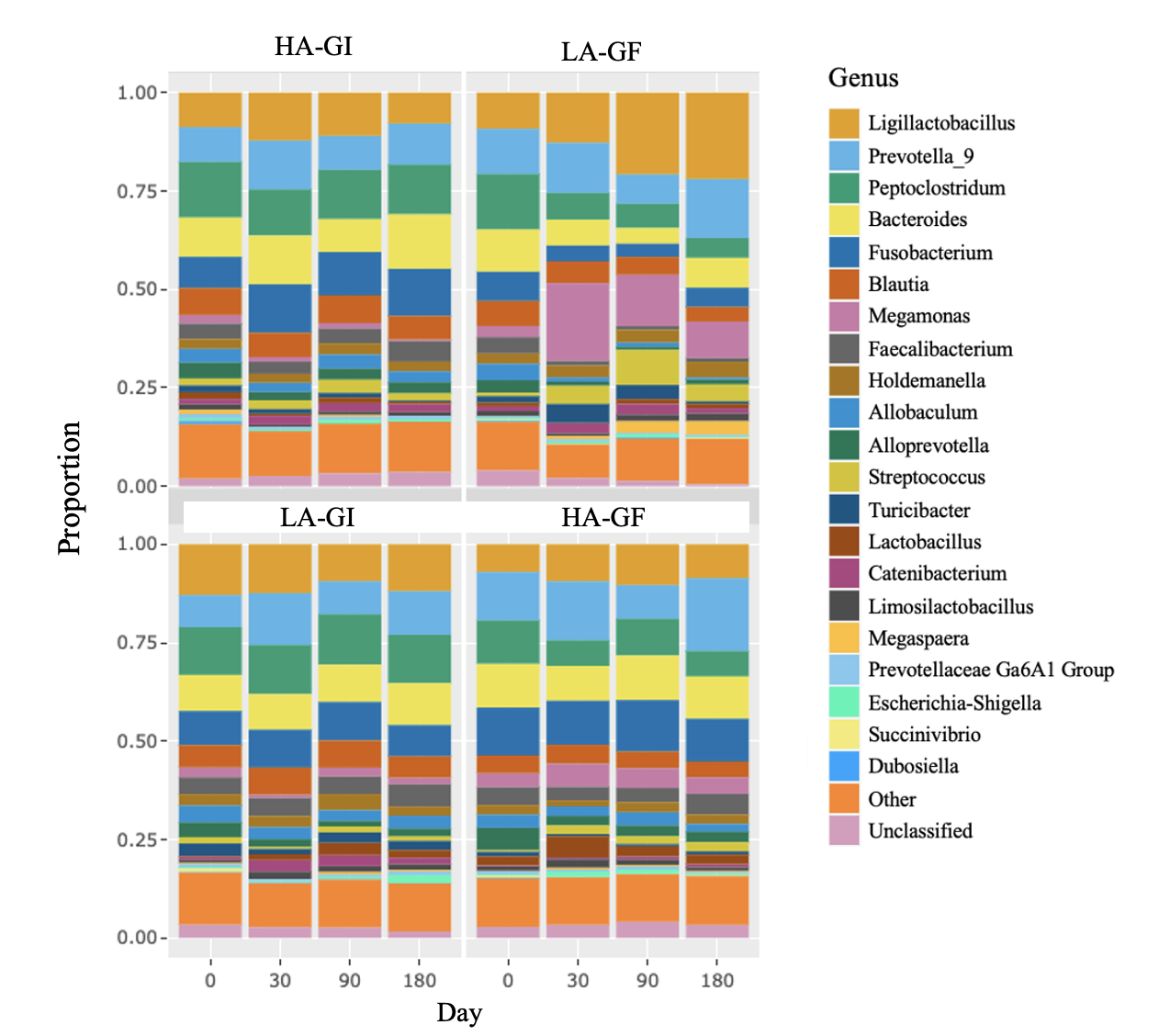


HA-GI: high animal protein grain-inclusive diet, LA-GF: low animal protein grain-free diet, LA-GI: low animal protein grain-inclusive diet, HA-GF: high animal protein grain-free diet
